# Inflammatory Bowel Diseases Are Associated with Polymyositis and Dermatomyositis—A Retrospective Cohort Analysis

**DOI:** 10.3390/medicina58121727

**Published:** 2022-11-25

**Authors:** Kassem Sharif, Niv Ben-Shabat, Muhammad Mahagna, Uria Shani, Abdulla Watad, Arnon D. Cohen, Howard Amital

**Affiliations:** 1 Department of Gastroenterology, Sheba Medical Centre, Tel-Hashomer 5265601, Israel; 2 Department of Medicine B, Zabludowicz Center for Autoimmune Diseases, Sheba Medical Center, Tel-Hashomer 5262100, Israel; 3 Sackler Faculty of Medicine, Tel-Aviv University, Tel-Aviv 6209813, Israel; 4 Section of Musculoskeletal Disease, NIHR Leeds Musculoskeletal Biomedical Research Unit, Leeds Institute of Molecular Medicine, University of Leeds, Chapel Allerton Hospital, Leeds LS7 4SA, UK; 5 Chief Physicians Office, Clalit Health Services, Tel Aviv 6209813, Israel; 6 Siaal Research Center for Family Medicine and Primary Care, Faculty of Health Sciences, Ben-Gurion University of the Negev, Beer Sheva 8410501, Israel

**Keywords:** inflammatory bowel diseases, epidemiology, polymyositis, dermatomyositis, ulcerative colitis, Crohn’s disease

## Abstract

*Background and Objectives*: Polymyositis and dermatomyositis (PM/DM) are classified as polygenic autoimmune diseases, whereas inflammatory bowel disease (IBD) is considered a polygenic autoinflammatory disease. In the literature, several cases exist reporting the co-occurrence of both conditions. At the molecular level, PM/DM and IBD share common genetic determinants including interferon regulatory factor and vitamin D receptor susceptibility loci. Accumulating evidence underline several indicators that confer poor prognosis in IBD, including antinuclear antibody positivity and the presence of other autoimmune diseases, therefore the aim of this study is to assess the association between these entities. *Materials and Methods*: This is a population-based retrospective study using data retrieved from a large electronic medical record in Israel, the Clalit health registry. The sample included PM/DM patients and age- and sex-frequency matched controls. The prevalence of IBD in PM/DM was compared between the two groups and logistic regression was applied to control for confounding variables. Predictors of IBD in patients with PM/DM were also explored. *Results*: Our study included 12,278 subjects with 2085 PM/DM patients and 10,193 age- and sex- frequency-matched controls. The incidence of IBD in patients with PM/DM was significantly higher even after controlling for various confounding variables (OR of 1.73, 95% CI 1.05–2.86, *p*-value = 0.033). Anti-nuclear antibodies (ANA) positivity was found to be an independent predictor for IBD diagnosis in patients with PM/DM (OR 3.67, 95% CI 1.01–13.36, *p* = 0.048). *Conclusion*: Our analysis reports an association between IBD and PM/DM. Such association could point towards a common pathophysiological background. Further research is needed to further describe the clinical courses and whether a unique therapeutic approach is warranted.

## 1. Introduction

Polymyositis (PM) and dermatomyositis (DM) are subtypes of idiopathic inflammatory myopathies characterized by progressive symmetric predominantly proximal muscle weakness [1]. Although closely related, polymyositis involves the endomysial layers of skeletal muscles where in contrast, dermatomyositis involves the perimysial layers of muscles with characteristic dermatological presentations [2]. The presence of autoantibodies and the inflammatory infiltration on histology points towards an autoimmune etiology [2]. PM and DM share many similarities in clinical manifestations. Earlier common diagnostic criteria included symmetric proximal muscle weakness, elevated serum muscle enzymes, myopathic changes in electromyography, characteristic muscle biopsy abnormalities, and typical rash of dermatomyositis [3]. Several myositis specific autoantibodies have been identified including anti-synthetase autoantibodies; a group of close to eight autoantibodies which are present in 25–30% of patients with DM or PM [4,5]. Anti-Jo-1; being the most frequent antibody, correlates with disease activity supporting a role in disease mechanism [6]. Myositis-associated autoantibodies are also present in PM/DM including anti-SSA and anti Ro-52 autoantibodies which are typically present in other autoimmune diseases providing a possible explanation to the increased risk of concomitant autoimmune/autoinflammatory diseases [7,8].

Inflammatory bowel diseases encompass ulcerative colitis (UC) and Crohn’s disease (CD) both resulting in intestinal inflammation. The two entities are differentiated by their mucosal depth and gastrointestinal involvement of the bowel. UC mainly affects the colon whereas CD results in inflammation anywhere along the lining of the digestive tract [9]. Extraintestinal manifestation are seen in 25–40% of inflammatory bowel disease (IBD) patients involving the musculoskeletal system including peripheral, axial arthritis, and enthesitis, dermatological such as leukocytoclastic vasculitis among other manifestations [10], hepatopancreaticobilary, ocular, metabolic and renal system [11,12].

The improved genetic understanding of immune related diseases permitted the elucidation of immune system perturbations and the categorization of immune diseases to distinct groups on a spectrum with one end representing autoimmune conditions and the other representing autoinflammatory diseases [13]. While clinically distinct, UC and CD share many genetic markers with a genetically defined autoinflammatory component thus classified as polygenic autoinflammatory diseases [14]. In the same sense, DM and PM are considered organ specific classic polygenic polygenic autoimmune diseases [13].

The rationale behind the need to clarify whether IBD is associated with PM/DM is due to observations identifying indicators that could confer poor prognosis in patients with IBD, including Anti-nuclear antibodies (ANA) in DM patients [15], presence of mucocutaneus manifestation including esophageal rings being associated with primary sclerosing cholangitis in UC patients [16], and the worse outcome of ileal pouch disease [17], increased pancolitis and clinical severity in UC patients with concomitant autoimmune diseases [18].

The association of inflammatory myopathies and inflammatory bowel disease has been reported sporadically in the literature, in this study we aim to investigate the prevenance and the association of these entities in a population based retrospective cohort.

## 2. Materials and Methods

### 2.1. Ethical Approval

The study was approved by the Ethical Committee of Clalit Health Services (CHS), located at the Soroka Medical Center, Beer-Sheva, Israel. Ethical approval code–0212-17-COM2, Date—11 March 2021.

### 2.2. Sample and Design

The study was designed as a population-based retrospective study using data extracted from the electronic medical database of the CHS. All patients with a documented diagnosis of polymyositis (ICD-9-code 710.4) or dermatomyositis (ICD-9-code 710.3) made by a specialist between the years 2002 and 2019 were included. Each patient was matched with five controls according to date of birth, gender, and place of residence. Rates of recorded diagnoses of Crohn’s disease (ICD-9-code 555.9) and ulcerative colitis (ICD-9-code 556.*) were compared between the groups. Each patient was directly matched with 5 controls without a diagnosis of PM/DM. Each subgroup analysis was done in comparison to the subgroup’s specific matched controls.

### 2.3. Database

CHS is the largest health organization in Israel with more than 4.4 million ensured members, corresponding to over 50% of the current Israeli population. CHS has a centralized database collecting continuous medical and administrative data from various sources including hospital admissions, primary health care visit, specialists’ clinics and pharmacy dispenses. The data undergoes several logistic checks to ensure data validity by comparing diagnoses from various sources and was previously demonstrated to have high validity [19,20,21].

### 2.4. Study Variables

All variables were derived from the CHS electronic health records. Demographic variables included age, gender, ethnicity (dichotomized into Arab and Jewish), socioeconomic status (based on area of residence according to the Israeli national census) body mass index (BMI) at the nearest time to diagnosis, smoking (dichotomized into ever vs. never). Treatment with glucocorticoids, methotrexate, azathioprine, rituximab, and intravenous immunoglobulins (IVIG), was defined based on a dispense of such agent made after the diagnosis of PM/DM. Laboratory test values of C-reactive protein (CRP), and creatine phosphate kinase (CPK), were defined as the highest values measured after diagnosis of PM/DM or matching date for controls. Serology of anti-Jo1 and antinuclear antibody (ANA) was defined positive based on the cutoff of the central lab of the CHS which conducted these tests.

### 2.5. Statistical Analysis

Continuous variables were reported as mean +/− standard deviation and were compared using student t-test. Categorical variables were reported as percentages and were compared using Pearson’s chi-square test. The association between inflammatory bowel disease and polymyositis and dermatomyositis was examine using a univariate and a multivariate logistic regression model. All P-values were two-tailed, and the null hypothesis was considered true if *p* ≥ 0.05. All statistical analysis was done using SPSS software, version 26 (SPSS, IBM Corp., Armonk, NY, USA).

## 3. Results

A total of 12,278 were included in this report with 2085 PM/DM patients, and 10,193 age- and sex- frequency matched controls. In our sample, 7236 participants were females (58.9), mean age of the cases group was 40.3 years old versus 40.1 in the control group. Of the 2085 subjects, 528 were diagnosed with PM and 1557 diagnosed with DM. Basic characteristics of both the case and control groups were similar (Table 1). Markers regarding disease activity including CPK levels, and CRP levels were significantly higher in patients with PM/DM. Concerning treatment, 67.6% of patients received glucocorticoids as part of their treatment plan, 15.3% received methotrexate, 9.2% were on azathioprine, 6.1% received IVIG, and in 2.9% received Rituximab. In PM/DM subjects, 34.5% had positive Anti-Jo-1 as compared to 8.6% in controls, *p* < 0.001. Basic characteristics including age, sex, SES distribution, BMI, smoking status are presented in Table 1.

Exploring the association between the factors of interest, the incidence of IBD was significantly higher in patients with PM/DM (OR 1.66, 95% CI 1.01–2.73, *p* = 0.045). Upon adjustment to various confounding factors including age, sex, ethnicity, SES, smoking, and BMI this association remained significant with OR of 1.73, 95% CI 1.05–2.86, *p*-value = 0.033. Given the small number of patients who developed IBD, the association between the disease subtypes (i.e., UC and CD) was not significant however trended positivity with an increased prevalence of PM/DM with both UC and CD (OR 1.53, 95% CI 0.75–3.12, *p* = 0.241, and OR 1.63, 95% 0.85–3.14, *p* = 0.142, respectively) (Table 2).

Patients with PM/DM were positive for myositis specific anti-Jo-1 and myositis non-specific autoantibodies including ANA (*p* < 0.001). When exploring predictors for developing IBD in PM/DM patients, ANA positivity was significantly associated with IBD diagnosis (OR 3.67, 95% CI 1.01–13.36, *p* = 0.048), other predictors are presented in Table 3.

## 4. Discussion

The results of this study support an association between IBD and PM/DM. In this report, there is an increased incidence of IBD in patients with PM/DM even after controlling for various confounding variables. Moreover, ANA positivity was a predictor for a diagnosis of IBD in the PM/DM subgroup. This is the first study to examine the relationship between these conditions in a large population-based study.

The relationship between PM/DM and IBD appears to be intricate with reports as early as 1970s hinting towards an association between the two conditions describing skeletal muscle involvement with granuloma formation in a patient with Crohn’s disease [22]. Across the years, several reports accumulated with cases revealing vasculitidic myositis, granulomatous myositis, myopathy, and dermatomyositis, conditions presenting with characteristic elevation of CPK, electromyographic and histological findings in patients diagnosed with either UC and CD [22,23,24,25]. Thus, it was concluded that myositis could present as an extraintestinal manifestation of IBD. Interestingly, in cases where the detection of PM/DM warranted further work-up for occult malignancy, investigations uncovered a diagnosis of UC [26], in other instances UC or CD developed after a disease latency [27,28].

Generally, patients with PM/DM may present with gastrointestinal manifestations with dysphagia being a rather common presentation, however lower gastrointestinal diseases involving the small and large intestine are less common. In a retrospective study on 48 PM/DM patients reporting lower gastrointestinal manifestations, only 3 had findings of oedematous hyperemic bowel wall with erosions and ulcerations in the lower gastrointestational tract. On histology, these lesions consisted of mucosal inflammation with ectasia. Mucosal inflammation affects peristalsis leading to both diarrhea and constipation perpetuating a vicious cycle ultimately resulting in further ulceration, and inflammation [29].

In our study, patients developed IBD after a diagnosis of PM/DM with a latency of several years. In the literature, while evidence is scant, PM/DM is considered to be a rare extraintestinal manifestation of IBD. It is known that about a quarter of patients develop extraintestinal manifestations prior to IBD onset [30], therefore it remains to investigated whether the cases of IBD following PM/DM are actually the latter being an extraintestinal manifestation predating the diagnosis of the former. When presenting as an extraintestinal disease, PM/DM is associated with the acute exacerbation of IBD with most cases improving upon treatment and control of the underlying gut disease [31].

A single nationwide cohort study was conducted exploring the incidence of PM/DM in UC patients using data from the Taiwanese national health insurance research database which included 3133 UC patients. In their analysis, the cumulative incidence of dermatomyositis was significantly higher in UC than that of control subjects (*p* = 0.026), however the cumulative incidence of polymyositis was comparable between the two groups (*p* = 0.596). Similar trends were observed after adjustment for confounding variables including concomitant rheumatologic conditions [32].

The mechanisms explaining the increased IBD risk in patients with PM/DM are not completely understood, however insights from genome wide association studies point towards a common denominator including the interferon-regulatory factors such as IRF5 rs4728142 and vitamin D receptor (VDR) rs2228570 [33,34,35].

From an immunopathology point of view, the inflammatory cell infiltrate in PM/DM is composed of both adaptive and innate immune cells including cytotoxic CD8+T-cells, CD4+ T-cells, macrophages, dendritic cells and B cells [4,36]. Such infiltrate has direct cytotoxic effect on muscle fibrils expressing major histocompatibility class MHC I molecules resulting in damage to the endomysium of skeletal muscles. Healthy differentiated muscle fibers do not express MHC I as contrasted to fibers in patients with myositis [36]. Increased expression of both MHC classes has been reported in PM/DM, however MHC Class I antigen expression is more frequently observed than class II [37]. In addition, the presence of autoantibodies and the fact that the major risk factor in Caucasian patients is HLA-DR3 point towards a role of MHC class II [38]. Similarly, IBD targeted studies indicate multiple independent associations with human leukocyte antigen (HLA) most consistently being HLA-DRB1 and HLA-DQB1 with reports indicating the association of HLA-C class I locus [39,40,41]. Together, this evidence points towards the polygenic nature of PM/DM and IBD, with the former being accepted as a polygenic autoimmune disease whereas the latter is considered a polygenic autoinflammatory condition [13].

This study has several strengths including the use of a population based large database health registry. Generally, the main limitation in the assessment of an association between PM/DM and IBD is the small subset of PM/DM patients developing an IBD disease, therefore the use of a nationwide wide cohort helps addressing this point. Despite this, our study has limitations including the reliance on registry data which may be problematic as some of the diagnoses could be entered incorrectly. However, various previous studies attest to the high validity of the diagnoses in our database, and the fact that diagnoses undergo logistic check to ensure validity by comparing data from various sources. While our database included laboratory findings and serological studies, it did not specify disease phenotypes. Moreover, in our study we grouped PM and DM as a group and UC and CD as another group to reasons eluded to earlier, while this allowed for better understating of the association between the two entitles, it might be that the combination of conditions may represent a unique entity different from either process alone Another concern might be that threshold for undergoing further investigations and initiating earlier consultation in patients diagnosed with one autoimmune disease might be lower, however in our study both cases and controls had similar lengths of latency.

In conclusion, our study demonstrates the increased incidence of IBD in patients diagnosed with PM/DM. ANA serological positivity was found to be a predictor for IBD in patients with PM/DM. The association between two rather uncommon diseases may point towards shared pathophysiological background. More research is needed to better elucidate the clinical course of patients with both comorbidities and whether the combination may represent a unique entity different from other individual entities.

## Figures and Tables

**Table 1:** Baseline chrematistics of the study population.

	Polymyositis (*n* = 528) ^a^	Controls (*n* = 2560) ^a^	*p* ^b^	Dermatomyositis (*n* = 1557) ^a^	Controls (*n* = 7633) ^a^	*p* ^b^
**Demographics**						
Age at diagnosis						
Mean ± SD	51.2 ± 18	50.9 ± 18	0.689	36.7 ± 22	36.4 ± 22	0.649
Median (IQR)	52.7 (38–65)	52.3 (38–65)	0.811	35.6 (17–56)	35.2 (16–56)	0.817
Female gender	333 (63.1)	1614 (63.0)	0.993	896 (57.5)	4393 (57.6)	0.996
Socioeconomic status			0.955			0.985
Low	139 (26.3)	660 (25.8)		400 (25.7)	1956 (25.6)	
Intermediate	321 (60.8)	1574 (61.5)		931 (59.8)	4556 (59.7)	
High	68 (12.9)	326 (12.7)		226 (14.5)	1121 (14.7)	
Ethnicity			0.926			0.853
Arab	117 (22.2)	572 (22.3)		329 (21.1)	1629 (21.3)	
Jewish	411 (77.8)	1988 (77.7)		1228 (78.9)	6004 (78.7)	
BMI (kg/m^2^)	28.1 ± 6	27.8 ± 6	0.553	26.2 ± 6	26.6 ± 9	0.050
Obesity (BMI > 30)	156 (30.4)	733 (29.9)	0.834	301 (22.3)	1537 (24.0)	0.193
Smoking (ever)	187 (35.4)	838 (32.7)	0.233	429 (27.6)	2241 (29.4)	0.152
Highest CRP (mg/L), mean ± SD	13.9 ± 37	9.5 ± 31	<0.001	10.5 ± 31	2.7 ± 16	<0.001
Highest CPK (U/L), mean ± SD	1761 ± 5394	250 ± 928	<0.001	1093 ± 5373	215 ± 932	<0.001
**Treatment**						
Glucocorticoids	413 (78.2)	-	-	996 (64.0)	-	-
Methotrexate	92 (17.4)	-	-	228 (14.6)	-	-
Azathioprine	76 (14.4)	-	-	115 (7.4)	-	-
IVIG	44 (8.3)	-	-	84 (5.4)	-	-
Rituximab	21 (4.0)	-	-	39 (2.5)	-	^-^
**Serology**						
Anti-Jo1	24 (4.5)	2 (0.1)	<0.001	38 (2.4)	1 (0.0)	<0.001
Antinuclear	157 (29.7)	192 (7.5)	<0.001	318 (20.4)	369 (4.8)	<0.001

^a^ *n* (%), mean ± SD; ^b^ *p*-value; Abbreviations: CPK, creatinine phosphate kinase; CRP, C-reactive protein; IVIG, intravenous immunoglobulins.

**Table 2:** Association of inflammatory myositis with IBD, logistic regression analysis.

	PM/DM (*n* = 2085)	Controls (*n* = 10,193)	*p*-Value
**IBD**			
Number of cases, *n* (%)			
Overall	21 (1.0)	62 (0.6)	
Diagnosed after PM/DM ^a^	11 (0.5)	24 (0.2)	
Diagnosed within 1-year difference from PM/DM ^a^	5 (0.2)	7 (0.1)	
Interval between diagnoses, years			
Mean (SD)	6.04 (6.1)	5.94 (5.4)	
Median (range)	5.12 (28.0)	4.11 (31.3)	
Odds ratio (95% CI)			
Unadjusted	1.66 (1.01–2.73)	ref	0.045
Age and sex adjusted	1.65 (1.01–2.71)	ref	0.048
Multivariate ^b^ adjusted	1.73 (1.05–2.86)	ref	0.033
Crohn’s disease			
Number of cases, *n* (%)			
Overall	12 (0.6)	36 (0.4)	
Diagnosed after PM/DM ^a^	7 (0.3)	12 (0.1)	
Diagnosed within 1-year difference from PM/DM	3 (0.1)	6 (0.1)	
Interval between diagnoses, years			
Mean (S.D)	4.21 (3.0)	5.95 (6.2)	
Median (range)	3.88 (9.2)	3.55 (31.3)	
Odds ratio (95% CI)			
Unadjusted	1.63 (0.85–3.14)	ref	0.142
Age and sex adjusted	1.62 (0.84–3.13)	ref	0.147
Multivariate ^b^ adjusted	1.75 (0.90–3.40)	ref	0.099
Ulcerative Colitis			
Number of cases, n(%)			
Overall	10 (0.5)	32 (0.3)	
Diagnosed after PM/DM ^a^	4 (0.2)	13 (0.1)	
Diagnosed within 1-year difference from PM/DM ^a^	2 (0.1)	1 (0.0)	
Interval between diagnoses, years			
Mean (S.D)	8.01 (7.9)	6.68 (4.3)	
Median (range)	6.97 (28.0)	5.50 (15.3)	
Odds ratio (95% CI)			
Unadjusted	1.53 (0.75–3.12)	ref	0.241
Age and sex adjusted	1.52 (0.74–3.09)	ref	0.251
Multivariate ^b^ adjusted	1.58 (0.77–3.24)	ref	0.212

^a^ index date for matched controls ^b^ adjusted for age, sex, ethnicity, socioeconomic status, smoking, body-mass-index. Abbreviations: DM, dermatomyositis; IBD, inflammatory bowel disease; PM, polymyositis.

**Table 3:** Predictors of IBD among patients with Polymyositis/Dermatomyositis.

	IBD (*n* = 21)	No IBD (*n* = 2064)	OR_age-and-sex_	95% CI	*p*-Value
Age at diagnosis (Mean ± SD)	48.6 ± 19.4	40.3 ± 22.4	1.09 ^a^	0.98–1.20	0.106
Female gender	14 (66.7)	1215 (58.9)	1.29	0.51–3.22	0.587
Low socioeconomic status	3 (14.3)	536 (26.0)	0.55	0.16–1.89	0.339
Arab ethnicity	3 (14.3)	443 (21.5)	0.74	0.21–2.60	0.643
Obesity	5 (23.8)	452 (24.5)	0.86	0.31–2.34	0.778
Smoking	7 (33.3)	609 (29.5)	1.13	0.44–2.92	0.801
Highest CPK (U/L), mean ± SD	1089 ± 1698	1291 ± 5415	1.00 ^b^	0.99–1.01	0.887
Highest CRP (mg/dL), mean ± SD	26.9 ± 39.1	11.4 ± 33.3	1.03 ^c^	0.99–1.07	0.057
Glucocorticoids	20 (95.2)	1389 (67.3)	8.36	1.09–63.80	0.041
Methotrexate	6 (28.6)	314 (15.2)	2.04	0.78–5.35	0.147
Azathioprine	6 (28.6)	185 (9.0)	3.66	1.39–9.63	0.008
IVIG	2 (9.5)	126 (6.1)	1.58	0.36–6.89	0.539
Rituximab	1 (4.8)	59 (2.9)	1.58	0.21–11.20	0.658
Anti-Jo1	1(4.8)	61 (3.0)	1.64	0.22–12.43	0.631
ANA	9 (42.9)	466 (22.6)	2.57	1.08–6.14	0.033

^a^ for every 5 years increment. ^b^ for every 50 U/L increment. ^c^ for every 5 mg/dL increment. Abbreviations: ANA, anti-nuclear antibodies; CPK, creatinine phosphate kinase; CRP, C-reactive protein, IBD, inflammatory bowel disease; IVIG, intravenous immunoglobulins.

## Data Availability

Data is not available publicly.
